# Tumor necrosis factor-related apoptosis-inducing ligand (TRAIL) deletion in myeloid cells augments cholestatic liver injury

**DOI:** 10.1038/s41598-024-52710-3

**Published:** 2024-01-25

**Authors:** Anuradha Krishnan, Nazli Begum Ozturk, Kaiyel A. Cutshaw, Maria Eugenia Guicciardi, Takashi Kitagataya, Kirsta E. Olson, Kevin D. Pavelko, William Sherman, Alexander Q. Wixom, Nidhi Jalan-Sakrikar, Michelle Baez-Faria, Florencia Gutierrez, Gregory J. Gores

**Affiliations:** 1 Division of Gastroenterology and Hepatology, Mayo Clinic, 200 First Street SW, Rochester, MN 55905 USA; 2 Department of Immunology, Mayo Clinic, Rochester, MN USA; 3 Department of Quantitative Health Sciences, Mayo Clinic, Rochester, MN USA

**Keywords:** Immunology, Gastroenterology

## Abstract

Ductular reactive (DR) cells exacerbate cholestatic liver injury and fibrosis. Herein, we posit that tumor necrosis factor-related apoptosis-inducing ligand (TRAIL) emanates from recruited macrophages and restrains DR cell expansion, thereby limiting cholestatic liver injury. Wild type (WT), *Trail*^*fl/fl*^ and myeloid-specific *Trail* deleted (*Trail*^*Δmye*^) C57BL/6 mice were exposed to DDC diet-induced cholestatic liver injury, which induced hepatomegaly and liver injury as compared to control diet-fed mice. However, parameters of liver injury, fibrosis, and inflammation were all increased in the *Trail*^*Δmye*^ mice as compared to the WT and *Trail*^*fl/fl*^ mice. High dimensional mass cytometry indicated that cholestasis resulted in increased hepatic recruitment of subsets of macrophages and neutrophils in the *Trail*^*Δmye*^ mice. Spatial transcriptomics analysis revealed that the PanCK^+^ cholangiocytes from *Trail*^*Δmye*^ mice had increased expression of the known myeloid attractants *S100a8, Cxcl5, Cx3cl1,* and *Cxcl1.* Additionally, in situ hybridization of *Cxcl1,* a potent neutrophil chemoattractant, demonstrated an increased expression in CK19^+^ cholangiocytes of *Trail*^*Δmye*^ mice. Collectively, these data suggest that TRAIL from myeloid cells, particularly macrophages, restrains a subset of DR cells (i.e., *Cxcl1* positive cells), limiting liver inflammation and fibrosis. Reprogramming macrophages to express TRAIL may be salutary in cholestasis.

## Introduction

Cholangiocytes, the specialized epithelial cells that line the bile ducts, account for 4 to 5% of the total liver cell population and perform unique physiological functions related to the modification and secretion of bile and the elimination of xenobiotics and drug metabolites^1,2^. Various injurious stimuli can lead to cholangiocyte dysfunction and biliary tract injury, resulting in disease states collectively referred to as cholangiopathies. Cholangiopathies are characterized by impaired bile formation or bile flow, hepatic inflammation, and fibrosis^2,3^. A prominent feature of cholangiopathies is the presence of ductular reactive (DR) cells, typically arising in the peri-biliary region, but which can extend into the hepatic parenchyma^2,4^. DR cells, which retain a cholangiocyte-like phenotype, are assembled into irregular duct-like structures that occur in close association with immune cells and activated myofibroblasts^5^. In human liver diseases the magnitude of DR cells correlates with the severity of liver fibrosis^6^. Aberrant signaling between DR cells, neighborhood immune cells and myofibroblasts occurs in cholestatic liver injury thereby potentiating inflammation and fibrosis. Macrophages are the predominant immune cell population in the DR cell niche^7^. However, they display conflicting roles in liver injury and repair, either contributing to disease mitigation or to its progression^7,8^. Understanding the nature of the ductular reaction in cholangiopathies and the contextual relationship between DR cells and macrophages would help clarify their contribution towards disease progression.

Tumor Necrosis Factor Related Apoptosis Inducing Ligand (TRAIL), encoded by the gene *Tnfsf10*, is the ligand to a canonical death receptor termed as TRAIL Receptor (TR) that is involved in pro-inflammatory and pro-apoptotic signaling^9^. TRAIL has two pro-apoptotic receptors in humans, TR1 known as death receptor 4 (DR4) and TR2 known as death receptor 5 (DR5), whereas mice have only one TR (mTR). The binding of TRAIL to TRs triggers either a canonical pro-apoptotic pathway resulting in cell death, or an alternative non-canonical signaling pathway that results in the activation of the NF-κB pathway which leads to inflammatory gene expression^10^. Interestingly, we have reported that germline deletion of mTR in the *Mdr2*^*−*/*−*^ mice, a well-established model of cholestatic liver disease, augments the ductular reaction, macrophage accumulation, and hepatic fibrosis^11^. Further, expansion of the DR cell population occurred in the context of decreased apoptosis without alterations of DR cell proliferation rates. Hence, pro-apoptotic TRAIL signaling in DR cells limits their abundance and mitigates cholestatic liver injury. TRs are ubiquitously expressed by all cells, whereas TRAIL, the only known ligand for the TR, is expressed primarily by immune cells, including macrophages^12^. The liver is populated by resident macrophages, also known as Kupffer cells, and recruited bone marrow-derived macrophages (BMDMs) derived from circulating monocytes^13,14^. Notwithstanding their origin, macrophages display a spectrum of activation states that are tissue and context specific^15,16^. Germline deletion of mTR in the *Mdr2*^*−/−*^ mice promoted the accumulation of a subset of alternately activated macrophages that were CD206^+^MERTK^+^LGALS3^+^. Moreover, MERTK^+^ macrophages also expressed TRAIL, suggesting that TRAIL originating from macrophages limited the expansion of DR cells^11^. Thus, we posited that macrophage-derived TRAIL restrains DR cell expansion and limits fibrosis by inducing DR cell apoptosis. Herein, we employed the 3,5-diethoxycarbonyl-1,4-dihyrocollidine (DDC) diet-induced cholestatic liver injury in mice that were deficient in TRAIL in the myeloid cell compartment. The results suggest that deletion of TRAIL in the myeloid cells is sufficient to augment liver injury and increase DR cell expansion and hepatic fibrosis. Myeloid TRAIL appears to restrain a DR cell subpopulation, providing insight into the mechanism governing the abundance of DR cells in cholestasis. Reprogramming the intrahepatic, myeloid cell population to express TRAIL may be a salutary therapeutic strategy in cholestatic liver injury.

## Results

### Deletion of *Tnfsf10 (Trail)* in myeloid cells augments liver injury in DDC diet-induced cholestasis

**Figure 1:**
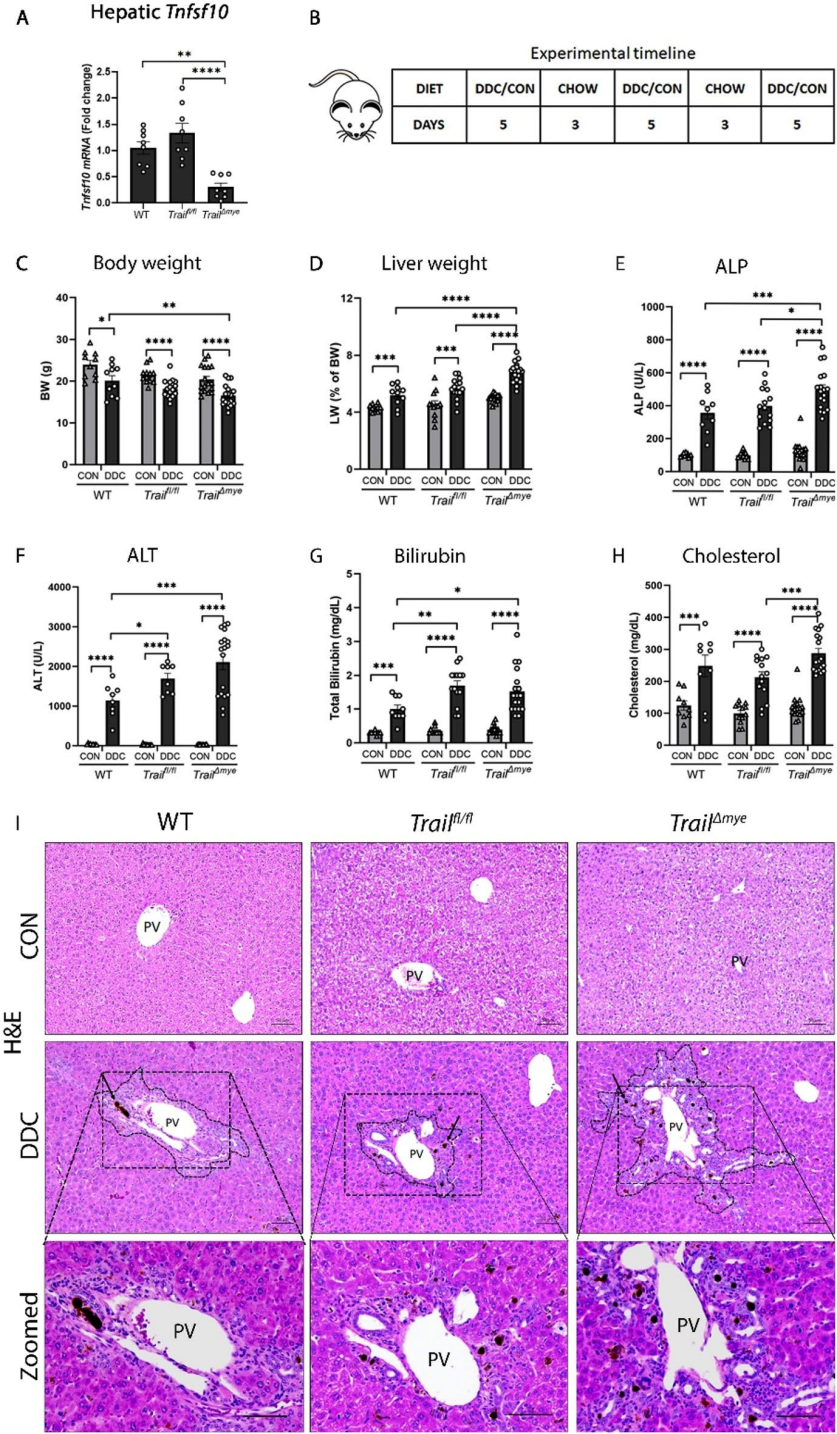
Deletion of TRAIL in myeloid cells augments liver injury and portal inflammation. (**A**) *Tnfsf10* (TRAIL) mRNA was decreased in the liver of the *Trail*^*Δmye*^ mice as determined by quantitative real-time PCR. (**B**) Schematic of the experimental timeline illustrating the intermittent feeding schedule with alternating days of DDC/control and chow diet. Morphological attributes and clinical features of liver injury and malfunction are graphically presented (**C**) Body weight, (**D**) liver weight expressed as percent of body weight, (**E**) ALP, (**F**) ALT (**G**) total bilirubin, and (**H**) cholesterol (*p < 0.05, **p < 0.01, ***p < 0.005, ****p < 0.001). (**I**) H&E-stained liver sections showing protoporphyrin (black arrows) in obstructed bile ducts. Dashed lines encompass the area of liver injury and inflammation in the DDC-fed mice. Zoomed images show the extent of injury and inflammation around the portal vein. *PV* Portal vein. Scale bar = 50 µm.

Intrahepatic expression of *Tnfsf10*, the gene for TRAIL, was significantly diminished in *Trail*^*∆mye*^ mice as compared to WT or *Trail*^*fl/fl*^ mice (Fig. 1A). Importantly, there was no difference in the hepatic gene expression of *Tnfsf10* between the WT or *Trail*^*fl/fl*^ mice. Age and gender-matched WT, *Trail*^*fl/fl*^ and *Trail*^*∆mye*^ mice were maintained on an intermittent feeding regime of the DDC diet as depicted in Fig. 1B. Body weights were decreased in all DDC diet-fed mice, as compared to the control diet-fed mice (Fig. 1C). Liver-body weight ratios were higher for all DDC-fed mice but were significantly elevated in the *Trail*^*Δmye*^ mice (Fig. 1D). Serum biochemical analysis indicated that while the DDC diet caused liver injury in all experimental mice, parameters of cholestatic liver injury, viz., serum ALP, and ALT were increased in the *Trail*^*Δmye*^ mice (Fig. 1E,F). Total bilirubin was also increased in *Trail*^*Δmye*^ mice as compared to WT mice with cholestatic injury (Fig. 1G). Additionally, cholesterol levels were elevated in the cholestatic *Trail*^*Δmye*^ mice (Fig. 1H). There were no significant differences between male and female mice. Histological evaluation of H&E staining revealed bile ducts obstructed with protoporphyrin in the DDC-fed mice (Fig. 1I). Portal inflammation was apparent in all cholestatic mice but was more extensive in the *Trail*^*Δmye*^ mice as compared to the WT or the *Trail*^*fl/fl*^ mice (Fig. 1I). Taken together, these observations suggest that conditional deletion of *Trail* in the myeloid cell population exacerbates the clinical features of cholestatic liver injury and portal tract inflammation.

### Deletion of *Tnfsf10 (Trail)* in myeloid cells causes expansion of the ductular reaction and increased hepatic fibrosis in DDC diet-induced cholestasis

**Figure 2:**
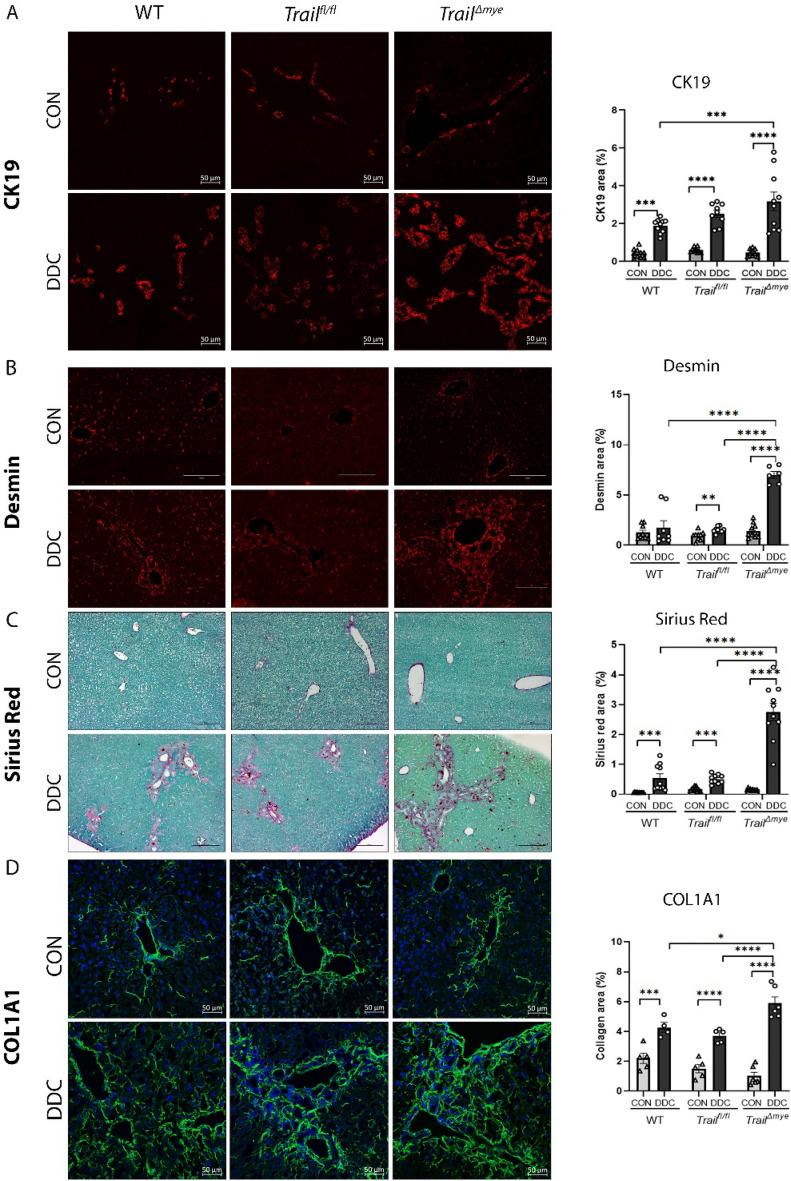
Ductular reaction and fibrosis are increased in cholestatic mice. (**A**) Representative images of FPPE liver tissue sections from WT, *Trail*^*fl/fl*^, and *Trail*^*Δmye*^ mice were immunostained for the cholangiocyte marker, CK19. Scale bar = 50 µm. Right panel displays the quantified data of digital image analysis of immunostained sections. (**B**) Representative images of FPPE mouse liver tissue sections immunostained for the myofibroblast marker, desmin. Scale bar = 150 µm. Digital image quantification is presented in the right panel. (**C**) Representative images of FPPE mouse liver sections stained for fibrosis with Sirius Red. Fast green was used as counterstain. Scale bar = 100 µm. Collagen was quantified using Sirius Red under plane-polarized light and is presented in the right panel. (**D**) Representative images of frozen mouse liver sections immunostained for the fibrillar collagen, COL1A1. The right panel shows the digital image quantification. (*p < 0.05, **p < 0.01, ***p < 0.005, ****p < 0.001).

**Figure 3:**
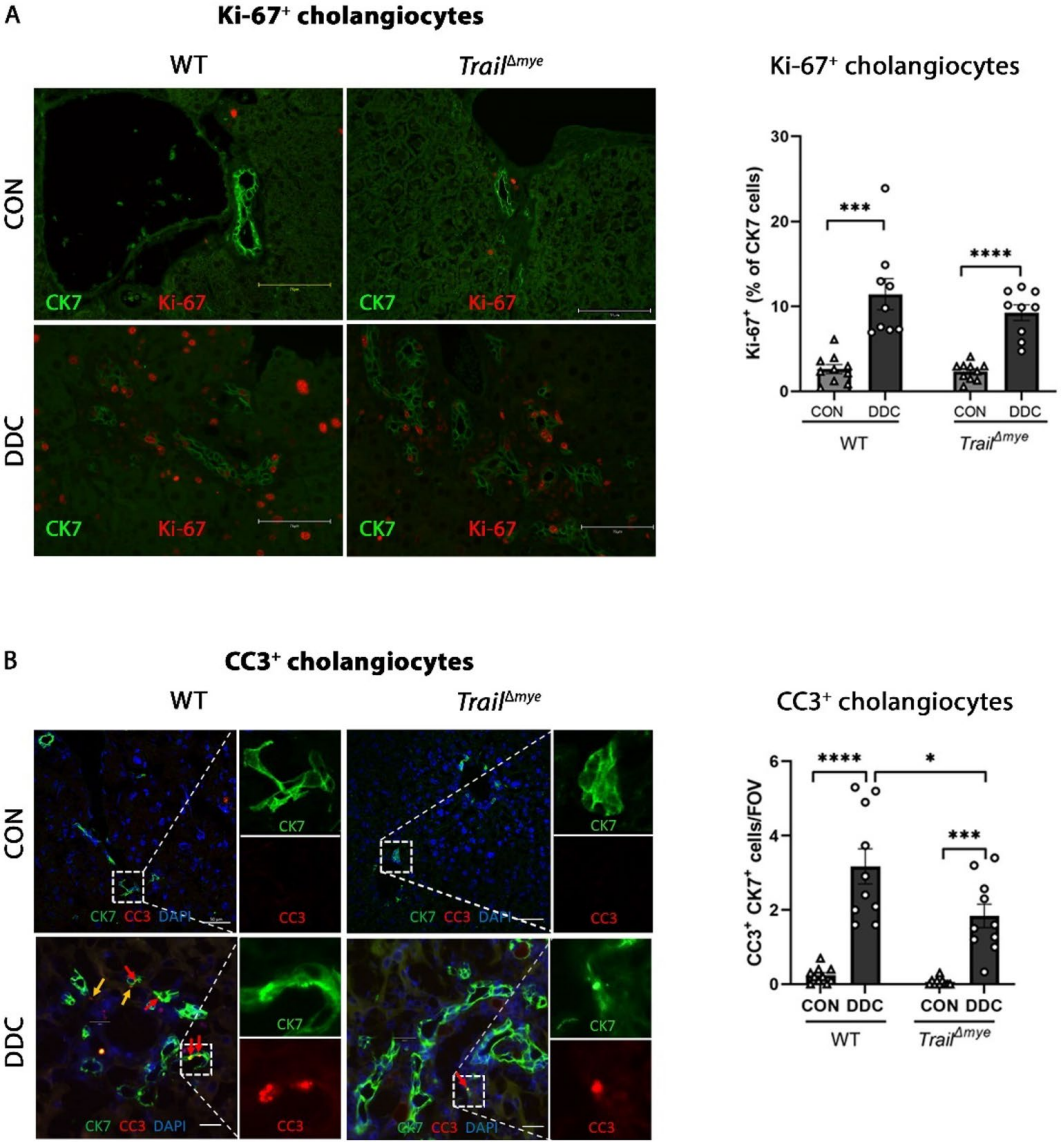
Cholangiocyte proliferation is similar whereas apoptosis is decreased in the cholangiocytes of cholestatic *Trail*^*Δmye*^ mice as compared to the WT mice. (**A**) Representative images of FPPE mouse liver tissue sections from WT and *Trail*^*Δmye*^ co-immunostained for the cholangiocyte marker, CK7 (green) and the proliferation marker, Ki-67 (red). Scale bar = 75 µm. Digital image quantification of CK7^+^Ki-67^+^ cholangiocytes is presented in the right panel. (**B**) Representative images of frozen mouse liver tissue sections co-immunostained for CK7 (green) and the apoptosis marker, cleaved caspase-3 (red). Red arrows point to CK7^+^ CC3^+^ cells, while yellow arrows point to CC3 staining in a different cell type. Scale bar = 50 µm. Digital image quantification of CC3 positive cholangiocytes is shown in the right panel. (*p < 0.05, ***p < 0.005, ****p < 0.001).

Liver sections were first immunostained for established markers of the ductular reaction, viz, CK19, SOX9^17^, and MIC1^18^. Upon quantification, we found that ductular reaction was increased in the DDC-fed WT and *Trail*^*fl/fl*^ and *Trail*^*Δmye*^ mice as determined by positivity for CK19 (Fig. 2A, Fig S1C), SOX9 (Fig S1D), and MIC1 (Fig S1E). Violin plots constructed from data enumerating CK19^+^ cells from individual fields of view demonstrated that the ductular reaction was expanded in the *Trail*^*Δmye*^ as compared to the DDC-fed WT mice (Fig S1C). Immunofluorescence analysis for well-established markers of myofibroblasts such as desmin (Fig. 2B), alpha smooth muscle actin (Fig S2B), and the extracellular matrix protein, fibronectin (Fig S2A), indicated an increased abundance of myofibroblasts in the DDC-fed *Trail*^*Δmye*^ mice and increased matrix deposition. Additionally, fibrosis was evaluated by Sirius red staining and by immunostaining for fibrillar collagen, COL1A1. Fibrosis was detected around obstructed bile ducts and in the areas adjoining the ductular reaction with fibrotic septa extending into the hepatic parenchyma in the *Trail*^*Δmye*^ mice livers (Fig. 2C). Analysis of Sirius red-stained sections under plane-polarized light confirmed that there was greater deposition of collagen in the injured livers of *Trail*^*Δmye*^ mice, as compared to the WT and *Trail*^*fl/fl*^ mice (Fig. 2C). Digital image analysis of immunostained liver sections for COL1A1 revealed a significant increase in the cholestatic *Trail*^*Δmye*^ mice, as compared to their WT or *Trail*^*fl/fl*^ counterparts (Fig. 2D). As increased accumulation of DR cells may be a consequence of enhanced proliferation or due to loss induced by TRAIL-mediated cell death by apoptosis, we evaluated both aspects by co-staining cholangiocytes for its’ marker, CK7, along with the proliferation marker, Ki-67, or the apoptosis marker, cleaved caspase 3 (CC3). CK7^+^Ki-67^+^ or CK7^+^CC3^+^ cells were enumerated. Whereas proliferation was similar in WT and *Trail*^*Δmye*^ DDC-fed mice (Fig. 3A), CK7^+^CC3^+^ cells were significantly diminished in the *Trail*^*Δmye*^ mice (Fig. 3B). Collectively, these data confirm that the DDC diet-induced cholestatic liver injury characterized by increased ductular reaction, and hepatic fibrosis in all mice. However, in the absence of TRAIL in the myeloid cells, these well-established features of liver injury are exacerbated. These results would suggest that TRAIL emanating from myeloid cells restrains and limits the expansion of the DR cells, as well as the myofibroblasts, likely by canonical pro-apoptotic signaling mechanisms.

### DDC diet-induced cholestasis causes changes in the liver immune phenotype

**Figure 4:**
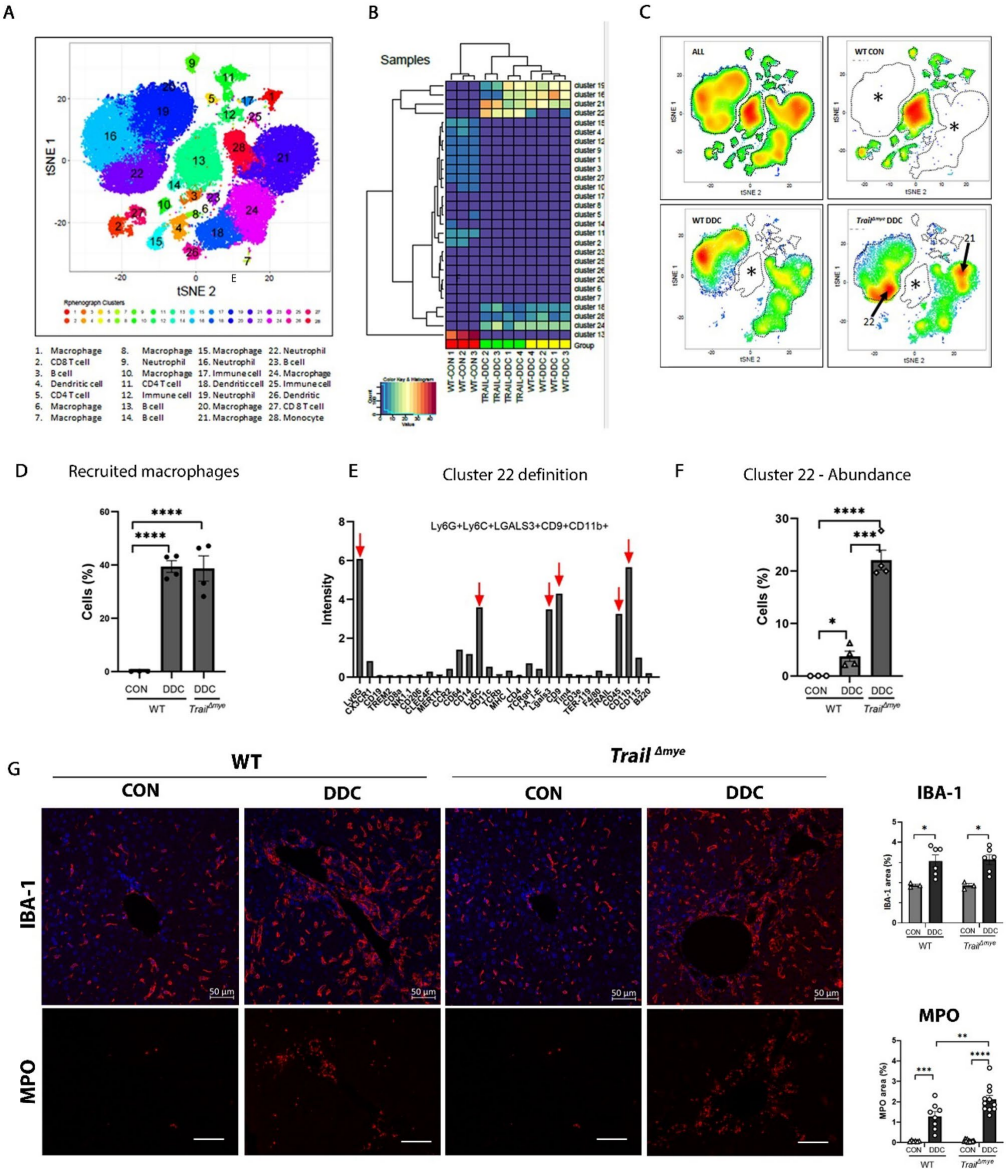
Immunophenotyping of intrahepatic leucocytes using mass cytometry. (**A**) t-SNE plot showing the spatial distribution of the 28 immune cell clusters resolved by unsupervised, non-linear dimension reduction. Similar cells cluster proximally, while dissimilar cells are located further apart. Each cluster is numbered and identified in the label below. (**B**) Hierarchical clustering heatmap of immune cell cluster abundance for all experimental mice is shown. Experimental groups (Label: *WT* Wild Type, TRAIL = *Trail*^*∆mye*^) are clustered together. (**C**) t-SNE plots of immune cell cluster abundance for pooled cells (All) and for each experimental group (WT CON, WT DDC, and *Trail*^*∆mye*^ DDC) is presented. Asterisks highlight the absence or the depletion of immune cell clusters within the experimental group. Arrows point to cell clusters that are abundant in the *Trail*^*Δmye*^ mice. (**D**) Total number of recruited macrophages (representing the sum of clusters 21, 24, and 28) is graphically presented. These clusters were distinguished by the presence of markers, Ly6C and CX3CR1. (**E**) The mean intensity of all markers of cluster 22 is graphed. Red arrows point to the most dominant markers used to identify the cluster immune phenotype as neutrophils. (**F**) The abundance of cluster 22 for each experimental group is quantified. (**G**) Representative images of FPPE mouse liver sections immunostained for the macrophage marker, IBA-1 (in red) (upper panel; scale bar = 50 µm) and the neutrophil marker, MPO (lower panel; scale bar = 100 µm) are shown along with the corresponding, quantified data on the right. (*p < 0.05, **p < 0.01, and ***p < 0.005, ****p < 0.001).

To gain a better understanding of the different immune cell populations that may contribute to cholestatic injury, we used the unbiased high dimensional platform, time-of-flight mass cytometry (CyTOF), to analyze total intrahepatic leukocytes isolated from WT mice fed either control or DDC diet, and from *Trail*^*Δmye*^ mice fed a DDC diet. The panel consisted of thirty cell surface markers that differentiated the lymphoid and myeloid immune cell compartments (Table 1). Twenty-eight distinct clusters of phenotypically related cells were distinguished based on the expression profiles of all markers from the pooled cells of all animals and visualized by a t-SNE plot (Fig. 4A). These included four B cell clusters (3, 13, 14, 23), two CD4^+^ T cell clusters (5, 11), two CD8^+^ T cell clusters (2, 27), three dendritic cell clusters (4, 18, 26), ten monocyte/macrophage clusters (1, 6, 7, 8, 10, 15, 20, 21, 24, 28), four neutrophil clusters (9, 16, 19, 22) and three smaller clusters of immune cells that were strongly immunogenic, as confirmed by CD45 positivity, but did not carry established makers for known cell lineages (12, 17, 25). The signal intensity of each marker and the resulting distinguishing signature of each cluster is provided (Fig S3A,B). The heatmap of cellular abundance of the clusters in individual mice (Fig. 4B) confirmed that the immune cell profiles of each experimental group were phenotypically distinct and clustered together based on their genotypes and on the injury model.

**Table 1:** List of antibodies and clones used for mass cytometry.

Antigen	Clone	Company	Catalog number	Label
B220	RA3-6B2	FDM	3176002B	176Yb
CCR2	475301	NOVUS	MAB55381-100	156Gd
CD115	AFS98	Biolegend	135521	174Yb
CD11b	M1/170	FDM	3172012B	172Yb
CD11c	N418	FDM	3142003B	142Nd
CD14	SA14-2	Biolegend	123321	165Ho
CD19	6D5	FDM	3166015B	166Er
CD206	C068C2	Biolegend	141702	151Eu
CD3e	145-2C11	FDM	3152004B	152Sm
CD4	RM4-5	FDM	3145002B	145Nd
CD45	30-F11	FDM	3089005B	089Y
CD64	290322	R&D	MAB20741	160Gd
CD8a	53–6.7	FDM	3168003B	168Er
CD9	MZ3	Biolegend	124802	147Sm
CLEC4F	–	R&D	AF2748	153Eu
CX3CR1	SA011F11	FDM	3164023B	164Dy
F4/80	BM8	FDM	3159009B	159 Tb
LGALS3	202213	R&D	MAB1197	141Pr
Ly6C	HK1.4	Biolegend	128039	175Lu
Ly6G	1A8	Biolegend	127637	161Dy
MERTK	108928	R&D	MAB5912	155Gd
MHC I	28–14-8	FDM	3144016B	144Nd
MHC II	M5/114.15.2	Biolegend	107637	150Nd
NK1.1	PK136	FDM	3170002B	170Er
TCR γ∆	GL3	Biolegend	118101	148Nd
TCRβ	H57-597	FDM	3143010B	143Nd
TER-119	TER-119	FDM	3154005B	154Sm
TIM4	RMT3-23	Biolegend	130002	149Sm
TRAIL	N2B2	Biolegend	109302	169Tm
TREM2	237920	R&D	MAB17291	167Er

Cholestasis caused profound changes in the immune cell profile of WT and *Trail*^*Δmye*^ mice as evidenced by the representative t-SNE plots of cell density for each group (Fig. 4C). Notably, B cells were the predominant immune cell type in WT mice reared on control diet, accounting for a total of 46.8% of the total intrahepatic immune population, while macrophages, dendritic cells and T cells accounted for 16.3%, 5.7%, and 19.85% respectively (Fig S4A). By contrast, macrophages were the dominant immune cell population in both the WT and *Trail*^*Δmye*^ mice fed the DDC diet, with total lymphocytes accounting for only 2.0% and 3.5%, respectively (Fig. 4C, Fig S4A).

A total of 15 markers (F4/80, CD11b, CLEC4F, Ly6C, CCR2, CXC3R1, CD64, CD14, MERTK, LGALS3, CD9, CD64, TREM2, TIMD4, and CD115) were used to characterize macrophage subsets (Table 1). Remarkably, the macrophage subsets in the control group differed significantly in their profile from the DDC-fed mice. Clusters 1, 6, 7, 8, 10, and 15 seen predominantly in the control diet-fed WT mice were absent in the DDC-diet fed mice (Fig. 4A,C). These clusters were defined by the presence of the macrophage marker F4/80, and varying co-expression levels of CD11b, MERTK, LGALS3, TIM4, CD206, and CLEC4F, known to be associated with the resident Kupffer cell populations (Fig S3B, S4A). Macrophage clusters (21, 24, 28) abundantly present in the DDC-fed WT and *Trail*^*Δmye*^ mice (Fig S4A) were virtually absent in the control group accounting for only 0.1% of the total immune population. Importantly, all three clusters expressed CX3CR1 and Ly6C (Fig S3B) indicating that these were monocyte-derived recruited macrophages that were greatly expanded in the cholestatic mice (Fig. 4D). These data indicate that the increase in macrophages observed in the DDC-fed mice is due to an increase in recruited cells, as opposed to proliferation of resident macrophages.

Similarly, unique neutrophil cluster profiles were seen in the control diet-fed mice versus the DDC diet-fed mice. Cluster 9 was observed only in the control diet group, whereas clusters 16, 19, and 22 were observed in both WT and *Trail*^*Δmye*^ mice on DDC diet (Fig. 4A,C, S4A). Specific subsets were differentially expressed between the two genotypes (Fig S4A). Clusters 16 and 19 predominated in WT DDC mice (Fig S4A), whereas cluster 22, bearing a classic neutrophil signature (Fig. 4E) was significantly upregulated in the *Trail*^*Δmye*^ mice (Fig. 4F). To further confirm our results, we next performed immunostaining for macrophages with the pan-macrophage marker, ionized calcium-binding adaptor molecule (IBA-1)^19^ and for neutrophils with myeloperoxidase (MPO). The results demonstrated a greater increase in IBA-1 macrophages in all DDC-fed mice as compared to the control diet-fed WT mice (Fig. 4G), while MPO was increased in the DDC-fed *Trail*^*Δmye*^ mice versus the DDC-fed WT mice (Fig. 4G). Collectively, these data suggest that the DDC diet promoted an influx of myeloid cells comprising of macrophages and neutrophils, although the subsets of these populations were present in varying proportions in the WT and *Trail*^*Δmye*^ mice.

**Figure 5:**
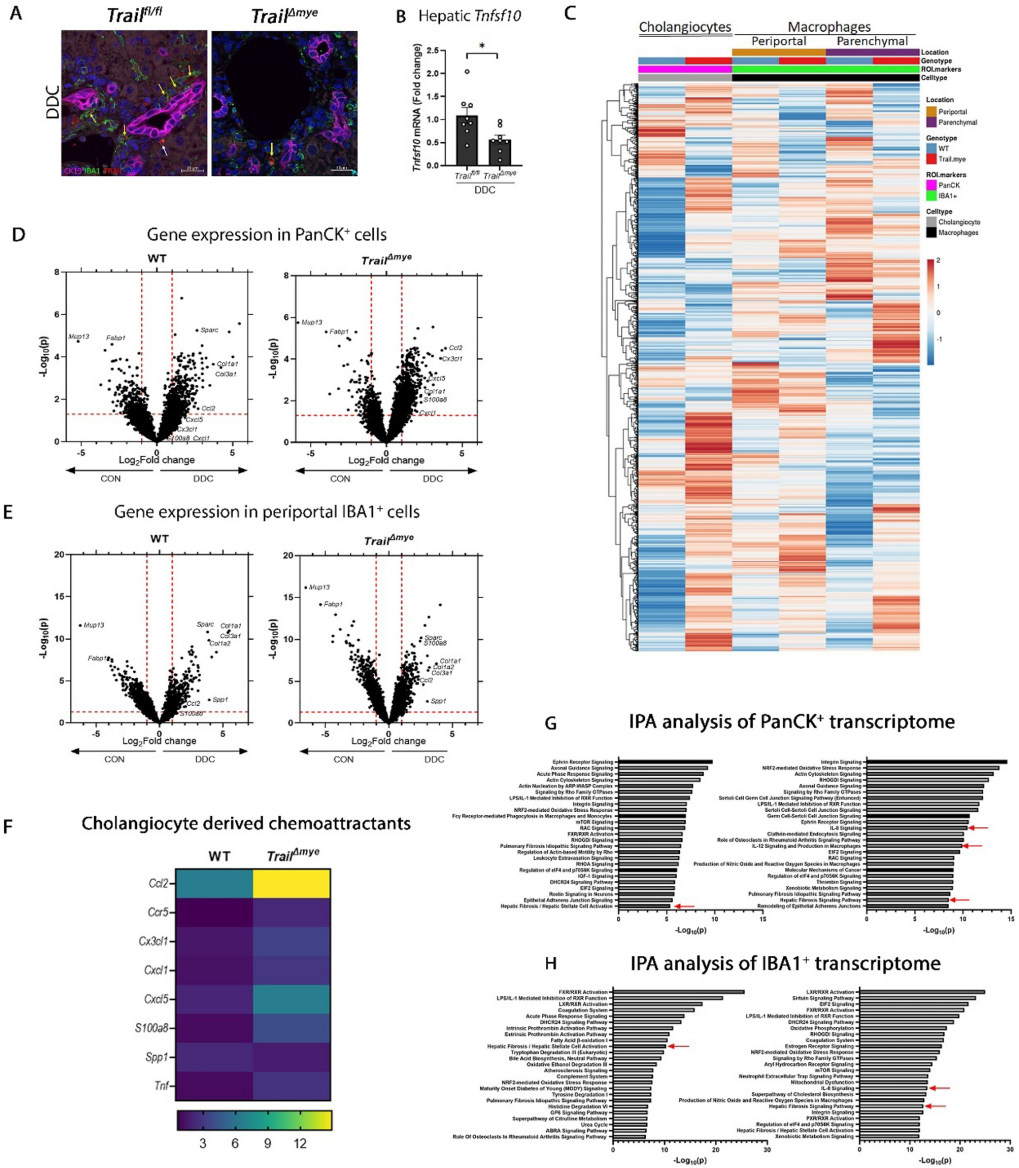
Diminished expression of TRAIL in cholestatic *Trail*^*Δmye*^ mice facilitates the retention of DR subsets. (**A**) Representative images of FPPE liver tissue sections of *Trail *^*fl/fl*^ and *Trail*^*Δmye*^ mice immunostained for the cholangiocyte marker, CK19 (magenta), pan-macrophage marker, IBA-1 (green), TRAIL (red), and nuclei (DAPI, blue). Yellow arrows point to TRAIL present in IBA1^+^ macrophages adjacent to cholangiocytes. White arrow shows TRAIL in another cell type. Scale bar = 20 µm. (**B**) Hepatic gene expression of *Tnfsf10* (TRAIL) is shown in cholestatic *Trail*^*Δmye*^ mice. (**C**) Hierarchical clustering heatmap of differentially expressed genes in cholestatic WT and *Trail*^*Δmye*^ mice were identified using spatial transcriptomics and filtered for genes of interest with an unbiased approach. The heatmap represents 2110 genes that were thus identified. Gene expression in cholestatic mice was normalized to gene expression in their respective control diet-fed mice. Differentially expressed genes in cholangiocytes and macrophages in the periportal and parenchymal segments are shown. Color codes for cell type (Cholangiocyte = grey, Macrophages = black), ROI (PanCK = magenta, IBA-1 = green), genotype (WT = blue, *Trail*^*Δmye*^ = red), and location (periportal = brown, parenchymal = purple) are indicated in the first 4 rows of the heatmap. (**D**,**E**) Volcano plots for the PanCK^+^ cells and the IBA1^+^ cells highlight the upregulation of pro-fibrotic, pro-inflammatory, and chemoattractant genes with cholestatic liver injury. Red dashed lines indicate values of *p* < 0.05 on the Y axis and log_2_ fold change (-1 and 1) on the X axis. (**F**) Heatmap of select chemoattractant genes differentially expressed in the cholangiocytes of WT and *Trail*^*Δmye*^ mice with cholestatic injury is presented. Gene expression of DDC-fed mice is normalized to their respective control diet-fed mice. (**G**,**H**) IPA analysis of the transcriptome of PanCK ^+^ and IBA1^+^ segments show the top signaling pathways activated with DDC diet-induced cholestatic liver injury. Red arrows point to pathways involved in inflammation, and hepatic fibrosis.

To confirm that macrophages within the DR cell niche express TRAIL, we co-stained liver tissue sections for IBA-1^19^ and for TRAIL. IBA1^+^ TRAIL ^+^ macrophages were observed adjacent to and in close juxtaposition to cholangiocytes with DDC diet-induced cholestatic liver injury in the *Trail*^*fl/fl*^ mice (Fig. 5A). By contrast, IBA1^+^ TRAIL^+^ macrophages were diminished in the *Trail*^*∆mye*^ mice with cholestatic injury (Fig. 5A). Specificity of the staining was verified by the absence of TRAIL positivity in *Trail*^*−/−*^ mice (Fig S4B). Importantly, hepatic gene expression of *Tnfsf10,* was significantly decreased in the *Trail*^*∆mye*^ mice with cholestatic injury as compared to the *Trail*^*fl/fl*^ mice (Fig. 5B). Since it is likely that TRAIL may be expressed by other immune cells and to clarify the source of TRAIL expression, we performed flow cytometry for TRAIL in peripheral blood polymorphonuclear leukocytes (PMNs), intrahepatic macrophages, and intrahepatic neutrophils of WT mice on DDC diet (Fig S4C). Although TRAIL was observed in the intrahepatic macrophage fraction, there was minimal expression in the peripheral blood and intrahepatic neutrophils. Collectively, these data suggest that (1) cholestatic liver injury promotes the recruitment of neutrophils and macrophages into the liver, (2) the primary source of TRAIL in DDC diet-induced cholestasis is the macrophage compartment, and (3) that deletion of TRAIL in myeloid cells favored the persistence and retention of specific subsets of neutrophils and macrophages within the liver.

### DDC diet mediated a pro-inflammatory response from cholangiocytes and adjoining periportal macrophages

**Figure 6:**
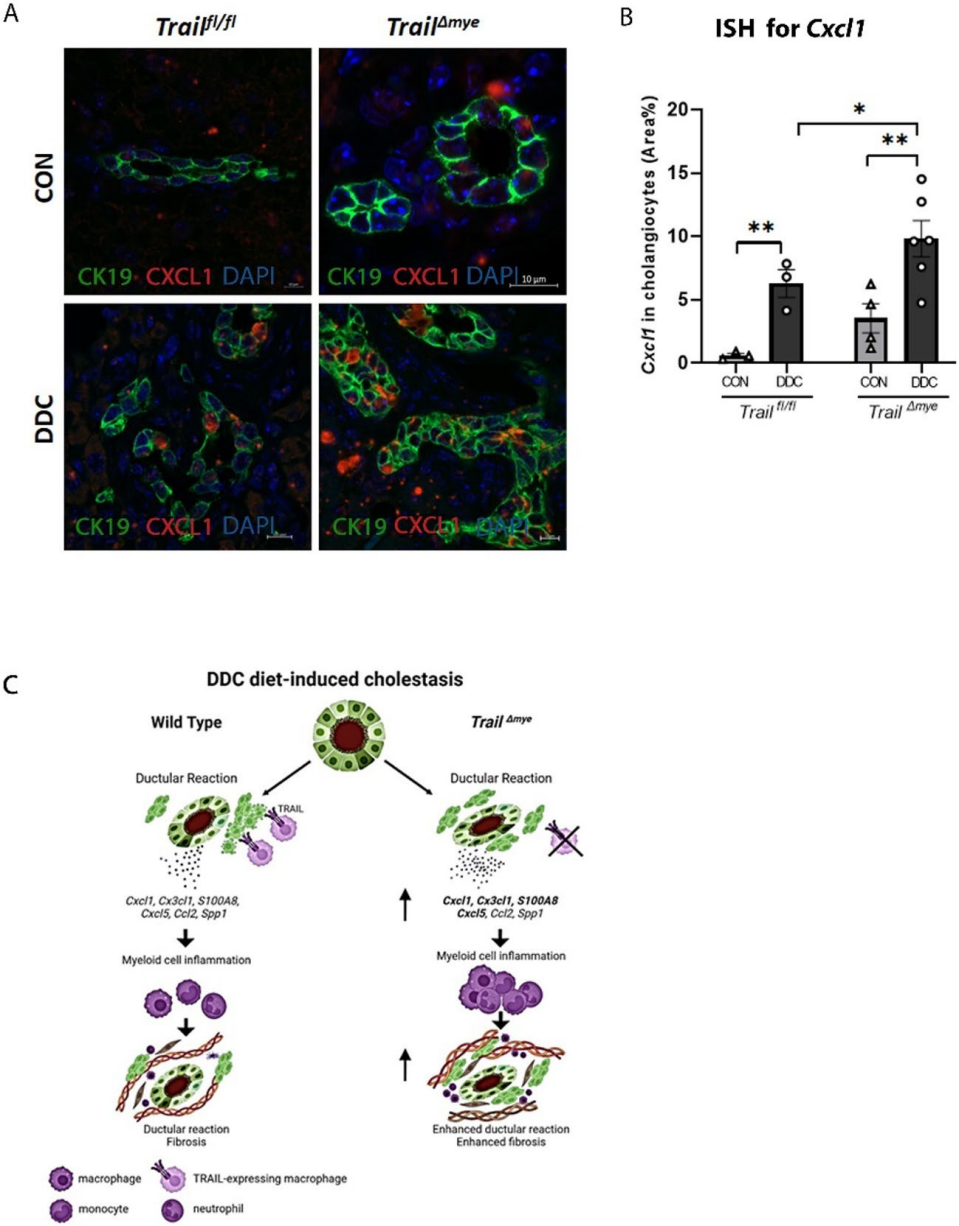
*Cxcl1* mRNA is significantly increased in the *Trail*^*Δmye*^ cholangiocytes. (**A**) Representative images of FPPE mouse liver tissue sections showing *Cxcl1* mRNA (red) expression by ISH, followed by immunostaining for CK19 (green) to define cholangiocytes. Scale bar = 10 µm. Some panels show zoomed images. (**B**) Cholangiocyte-specific *Cxcl1* mRNA was quantified (*p < 0.05, **p < 0.01). (**C**) A schematic depicting the differing outcomes of DDC diet-induced cholestatic liver injury in WT and *Trail*^*Δmye*^ mice. Depletion of TRAIL in myeloid cells results in the persistence of DR subsets, a source of pro-inflammatory chemoattractants. Neutrophils and fibroblasts accumulate in the liver leading to enhanced inflammation and increased fibrosis. The schematic was generated in Biorender.com.

To better resolve the gene expression profile of cholangiocytes that may promote the recruitment of myeloid cells into the periportal neighborhood, we performed spatially resolved transcriptomic analysis using FPPE liver tissue sections from WT and *Trail*^*Δmye*^ mice that were fed the control or DDC diet. Multiple regions of interest (ROIs) were delineated in periportal regions and in parenchymal regions. The morphology markers, PanCK and IBA1, were used to segment the cholangiocyte and macrophage populations (Fig S5A), thus allowing the capture of cell-specific gene expression. We identified 10,548 genes in greater than 10% of the ROIs. Principal component analysis (PCA) highlighted the wide variance in gene expression profiles between the WT and *Trail*^*Δmye*^ cholangiocytes, as well as the periportal and parenchymal macrophages (Fig S5B). Additionally, by adopting an unbiased approach of setting a filter for those genes having a high coefficient of variance (20% CV), 2110 genes of interest could be identified. Differential expression of these genes is depicted in a heatmap for the cholangiocyte, periportal and parenchymal macrophage segments (Fig. 5C). The data represent gene expression of DDC-fed WT and *Trail*^*Δmye*^ mice, normalized to the gene expression of their respective control diet-fed mice. Volcano plots for PanCK^+^ (Fig. 5D) and periportal IBA1^+^ (Fig. 5E) cells indicated that the DDC diet induced a pro-inflammatory and pro-fibrotic signature in WT and *Trail*^*Δmye*^ cholangiocyte and periportal macrophages. Of particular interest, gene expression of chemokines *S100a8*, *Ccr5, Tnf*, *Cxcl5, Cx3cl1,* and *Cxcl1* were upregulated in the cholangiocytes of *Trail*^*Δmye*^ mice fed DDC as compared to the cholestatic WT mice (Fig. 5F). In addition, Ingenuity pathway analysis (IPA) analysis indicated that IL-12 signaling was present among the top signaling pathways in the cholangiocytes, while IL-8 signaling was seen in both the PanCK^+^ cholangiocyte and the periportal IBA1^+^ macrophage segments in the *Trail*^*Δmye*^ (Fig. 5G,H). To confirm whether periportal cholangiocytes expressed neutrophil attractants, we performed in situ hybridization (ISH) for *Cxcl1* mRNA. Indeed, the DDC diet-induced cholestatic injury resulted in an increased expression of *Cxcl1* mRNA in the *Trail*^*Δmye*^ mouse cholangiocytes compared to *Trail*^*fl/fl*^ mouse cholangiocytes (Fig. 6A,B). Within the IBA1^+^ parenchymal macrophages, no differences in transcriptomic signature could be discerned between the WT and the *Trail*^*Δmye*^ mice by volcano plots (Fig S5C) or by IPA analysis (Fig S5D). Collectively, these data suggest that the cholangiocytes in the *Trail*^*Δmye*^ mice were more pro-inflammatory and likely promoted the increased recruitment of myeloid cells into the intrahepatic environment.

## Discussion

The results of this study provide key mechanistic insights into the regulation of DR cells during a DDC diet-induced murine model of cholestatic liver injury. The principal findings of this study are as follows: (1) DR cell abundance was increased in all cholestatic mice (2) the increase in a subset of DR cells (*Cxcl1* positive) in *Trail*^*Δmye*^ DDC-fed mice was due to decreased apoptosis, as DR cell proliferation was not altered; (3) the immune landscape was altered in *Trail*^*Δmye*^ mice and notable for the presence of unique subsets of neutrophils; and (4) consistent with the increase in neutrophils in *Trail*^*Δmye*^ mice, DR cells expressed chemokines for neutrophils in greater abundance. These data imply that TRAIL expressing myeloid cells, particularly macrophages, preferentially eliminate those DR cells promoting neutrophil associated hepatic inflammation and is conceptualized in Fig. 6C. Therefore, TRAIL expressing macrophages are important for reducing inflammation during cholestasis.

The expanded ductular reaction in mice on the DDC diet is consistent with DR cell-driven inflammation. DR cells are well known to become “activated” and secrete inflammatory mediators^5,20^. Hence, to gain further insight into this process, we profiled the immune landscape of the liver employing mass cytometry. We identified an increase in myeloid cell populations including both macrophages and neutrophils in the *Trail*^*Δmye*^ mice. Nearly, all clusters of macrophages in the cholestatic mice bore the distinctive recruitment signature expressing Ly6C, CX3CR1, CD14, CD64, CCR2, and CD115, indicating that they were monocyte-derived macrophages. This observation is consistent with prior studies conducted by us and others, reporting an increase in monocyte-derived macrophages in cholestatic disorders^7,19,21^.

Interestingly, there were three clusters of neutrophils that were differentially expressed between the cholestatic WT and *Trail*^*Δmye*^ mice; Cluster 22, identified as classic neutrophils, was more predominant in the *Trail*^*Δmye*^ mice than in WT mice. Immunostaining for MPO, a neutrophil-specific enzyme, confirmed the increased accumulation of neutrophils in the cholestatic *Trail*^*Δmye*^ mice. An excessive accumulation of neutrophils is generally associated with tissue damage^22^. Interestingly, a recent study found that ductular reaction associated neutrophils (DRANs) was a hallmark of advanced liver disease and increased with liver disease progression^23^. DRANs were immobilized at the site of ductular reaction for prolonged periods, exhibited reduced phagocytic capacity and exacerbated hepatic injury through increased oxidative burst. Furthermore, in keeping with previous studies demonstrating accelerated tissue repair with clearance of neutrophils^24^, depletion of DRANs limited the expansion of ductular reaction, and ameliorated liver fibrosis and angiogenesis^23^. To determine the cellular source of the chemokines responsible for promoting neutrophil recruitment into the liver, we employed spatial transcriptomics. Indeed, cholangiocytes were a prominent source of potent neutrophil chemoattractants such as *S100a8, Cxcl1, Cxcl5,* and *Cx3cl1*^25^. This observation was confirmed by the increased expression of *Cxcl1* mRNA seen in mouse liver cholangiocytes of the cholestatic *Trail*^*Δmye*^ mice using the ISH technique. These data imply that TRAIL-expressing myeloid cells, particularly macrophages, may preferentially eliminate those DR cells involved in promoting neutrophil-associated hepatic inflammation. and therefore, are important for reducing inflammation during cholestasis.

Our data support the concept that TRAIL expressing-myeloid cells are protective in cholestatic liver injury. Specifically, in the DDC diet-induced cholestatic liver injury model, myeloid cell-derived TRAIL limits a population of activated cholangiocytes that express chemokines,which recruit neutrophils to the liver and may additionally be instrumental in clearing expended neutrophils. This interpretation of our data is also consistent with observations that TRAIL^+^ iNOS^+^ macrophages are critical in reducing inflammation in models of atherosclerosis^26^. Furthermore, TREM2^+^ macrophages have also been reported to be a negative regulator of inflammation in cholestatic liver injury by bile duct ligation and administration of alpha-naphthylisothiocyanate (ANIT)^27^. We have previously demonstrated that TRAIL was expressed by a MERTK^+^ macrophage subpopulation^11^. However, the precise characteristics of the subpopulation of macrophages expressing TRAIL in cholestasis remains to be further characterized and will require detailed future work. In summary, we provide evidence that TRAIL-TR signaling provides a mechanism for limiting the ductular reaction, a primary source of pro-fibrotic, and pro-inflammatory cues, thus keeping disease progression in check. Augmenting the population of TRAIL positive macrophages or reprogramming macrophages to express TRAIL may be salutary in cholestasis.

## Materials and methods

### Animals and induction of cholestatic liver injury

All animal procedures were approved by and performed in accordance with the Institutional Animal care and Use Committee (IACUC) of the Mayo Clinic, Rochester, MN, in compliance with the ARRIVE guidelines. All experimental mice were reared on a 12-h light–dark cycle and had ad-libitum access to food and water. Cholestatic liver injury was induced in age (6–8 weeks) and gender-matched, experimental mice by feeding a diet infused with diethoxycarbonyl-1,4-dihydrocollidine (DDC) at 0.1% (104242 GI, Dyet Inc., Bethlehem, PA, USA). The feeding regime consisted of three rounds of DDC diet (5 days each), interspersed with two rounds of standard rodent chow (3 days each; Rodent Diet 5056, Pico Lab diets), for a total of twenty-one days (Fig. 1B). Mice were euthanized on day 22 by carbon-di-oxide inhalation and body weights determined. Blood was drawn via cardiac puncture. The liver was flushed with PBS via the portal vein, isolated, and weighed. Subsamples of each lobe were immediately preserved in 10% buffered formalin (245–684, Fisher HealthCare), embedded in Optimum Cutting Temperature (OCT) compound, or snap-frozen for other downstream applications.

### Generation of the ***Tnfsf10***^***Δmye***^ mice

Cryopreserved sperm of C57BL/6N-*Tnfsf10*^*tm1a(KOMP)Wtsi*^ (Strain 050165) was acquired from the Knockout Mouse Project (KOMP) repository maintained by the Mouse Mutant Resource and Research Center (MMRRC) at the University of California (Davis, CA). In vitro fertilization (IVF) of C57Bl/6 oocytes and the generation of mutant mice was carried out by the Transgenic and Knockout Core facility of the Mayo Clinic (Rochester, MN). The presence of the mutant knock-out first allele (Fig S1A) was confirmed by genotyping (Mouse PCR protocol design ID:96621, MMRRC @ UCDavis) using DNA isolated from tail snips. Germline confirmed mice (n = 4) were crossed over two filial generations to generate mice homozygous for the mutant knock-out first allele (Fig S1B). In vivo recombinase breeding was performed to remove the built-in trapping casette (En2SA-IRES-pA) and the Neo casette (hbactP-Neo-pA) by breeding to Flippase-expressing mice (Strain 016226, B6N.129S4-*Gt(ROSA)26Sor*^*tm1(FLP1) Dym*^/J, Jackson Lab, Bar Harbor, ME) to generate the conditional knock-out mice (Fig S1B). Homozygous conditional knock-out mice were then crossed with the B6.129P2-*Lyz2*^*tm1(cre)Ifo*^/J mice (Strain #004781, Jackson Laboratory, Bar Harbor, ME) to generate the *Tnfsf10*^*Δmye*^ mice. For the purposes of this paper, these mice will be referred to as *Trail*^*Δmye*^. *Trail*^*Δmye*^ mice exhibited normal morphological features, were fertile and *inter se* mating displayed normal Mendelian ratios. Deficiency of mRNA for *Tnfsf10* in bone marrow-derived macrophages isolated as previously described^28^ was confirmed by PCR (Fig S1A) using total RNA isolated with RNeasy® Plus mini kit (Qiagen, Hilden, Germany) and reverse transcribed into cDNA using iScript cDNA synthesis kit (Bio-rad, Hercules, CA) (Fig S1A). WT mice and *Trail*^*fl/fl*^ littermates were used as controls.

### Serum biochemistry

Blood samples acquired via cardiac puncture were centrifuged at 8000 rpm for twenty minutes to separate out the serum. One hundred µl of serum was analyzed for alanine transferase (ALT), alkaline phosphatase (ALP), total bilirubin, and cholesterol using the mammalian liver profile rotor (500–7128; Abaxis, Union City, CA) on the Vetscan VS2 (Abaxis Inc, CA, USA).

### Quantitative real time PCR

Total RNA was isolated from 50–100 mg liver tissue samples using the TRIZOL protocol. 1000 ng of total RNA was used to transcribe cDNA using the iScript Biorad cDNA Synthesis Kit (Cat No. 1708891, BioRad, USA). Quantitative real time PCR was carried out on a Roche 480 (Roche Diagnostic Corporation) using Lightcycler 480 SYBR Green Master I (04707516001, Roche, Diagnostic). Fold change in gene expression of *Tnfsf10* was calculated using the 2^−ΔΔCt^ method normalized over the geometric mean of two housekeeping genes, *18S* and *Gapdh.* The primers used were: 18S Forward—5’-CTCAACACGGGAAACCTCAC-3’, Reverse—5’-CGCTCCACCAACTAGAACG-3’; *Gapdh—*Forward—5’-AGGTCGGTGTGAACGGATTTG-3’, Reverse—5’-TGTAGACCATGTAGTTGAGGTCA-3’; and for *Tnfsf10* Forward—5’-CCTCAGCTTCAGTCAGCACT-3’, Reverse—5’—AGCTGCCACTTTCTGAGGTC-3’.

### Histology and immunostaining of liver tissue sections

Processing, paraffin embedding, and sectioning of formalin preserved liver tissue samples, cryosectioning of OCT-embedded tissue samples, and hematoxylin–eosin staining was performed by the Biomaterials and Histomorphometry Core facility of the Mayo Clinic, Rochester, MN. 5 µm thick serial sections of formalin-preserved, paraffin-embedded (FPPE) tissue samples or cryosections were assessed for markers of ductular reaction (CK19, SOX9, MIC1-1C3), fibrogenesis and fibrosis (αSMA, desmin, fibronectin, Sirius Red, and COL1A1), cell proliferation (Ki-67), cell death (Cleaved Caspase 3/CC3) macrophages (IBA-1), neutrophils (MPO) and TRAIL using immunofluorescent methods that were previously described^11^. Details of antibody clones, dilutions, and types of sections used are provided in Table 2. Staining for all antibodies was optimized to ensure minimal background and verified for specificity using secondary antibodies alone. Quantification of stained area was performed under plane polarized light for Sirius Red using an inverted microscope (Nikon Eclipse TE300, Nikon, Japan) and the NIS Elements AR software (v 4.60.00, Nikon). For all other immunostaining assays, a minimum of ten fields of view (FOV) images were acquired per section on an EVOS M5000 or a confocal microscope (LSM980, Zeiss, Germany). Digital image analysis for CK19, MIC1, desmin, SOX9, fibronectin, αSMA, COL1A1, IBA-1, MPO, and *Cxcl1* was performed using the ImageJ software (NIH, US). CK7^+^Ki-67^+^ cholangiocytes and CK7^+^CC3^+^ cholangiocytes were manually counted in at least ten random FOV. Data are presented either as area percentage, as a percentage of CK7^+^ cells.

**Table 2:** List of primary antibodies used for immunostaining and flow cytometry.

Target antigen	Company	Catalog number	Host species	Method	Dilution
αSMA	Abcam	AB124964	Rb mAb	IF	1:1000
CC3	Cell signaling technologies	9661S	Rabbit mAb	IF	1:100
CK19	Cell signaling technologies	12434S	Rabbit	IF	1:100
CK7	Santa cruz biotechnology	SC-53263	Mouse	IF	1:100
Desmin	Thermo-Fisher	RB-9014-P0	Rabbit pAb	IF	1:200
Desmin	Cell signaling technologies	D93F5	Rabbit mAB	IF	1:100
Fibronectin	BD Biosciences	610077	Mouse	IF	1:100
IBA-1	Novus biologicals	100–1028	Goat pAB	IF	1:100
Ki-67	Cell signaling technologies	12202S	Rabbit pAb	IF	1:100
MIC1	Novus biologicals	NBP1-18961	Rat mAb	IF	1:100
MPO	R&D Systems	AF3667	Goat pAB	IF	1:100
SOX9	EMD Millipore	AB5535	Rabbit pAb	IF	1:100
TRAIL	Abcam	Ab2435-1	Rabbit pAB	IF	1:100
COL1A1	Abclonal	A1352	Rabbit pAB	IF	1:100

*IF* Immunofluorescence.

### Isolation of intrahepatic leukocytes

Intrahepatic leukocytes were isolated from WT and *Trail*^*∆mye*^ mice as outlined previously^11^. Briefly, the liver was weighed, minced, and digested with a liver dissociation enzyme mix (130–105-807, Miltenyi Biotech) in a gentleMACS™ Octo tissue dissociator. Dissociated cells were centrifuged at 300 g for 5 min at 4 °C. Red blood cells were lysed (155 mM ammonium chloride, 12 mM sodium bicarbonate, 0.1 mM ethylene diamine tetraacetic acid) on ice for 5 min. The remaining cells were filtered through a 70 $\upmu$ m cell strainer. Kupffer cells, recruited monocytes, and the macrophage fraction were collected from the interphase layer of a 25–50% Percoll density gradient^11^. The cell pellet containing the polymorphonuclear (PMN) and lymphocyte fraction was also collected. Both fractions were combined and washed twice with wash buffer (PBS with 10 mM EDTA and 0.5% BSA).

### Time-of-flight mass cytometry (CyTOF)

The intrahepatic immune cell population was spun down at 300 g for 5 min and resuspended in cell staining buffer (Fluidigm). A minimum of 3 million cells were stained with an antibody cocktail (Table 1) conjugated to lanthanide-based metal isotopes according to manufacturer guidelines (Fluidigm, San Francisco, CA). Custom conjugations were performed by the Mayo Clinic Hybridoma Core (Rochester, MN). Alternately, pre-conjugated antibodies were purchased directly from Fluidigm. Mass cytometry was performed at the Mayo Clinic Immune Monitoring Core (Mayo Clinic, Rochester, MN) on a Helios mass cytometry system (Fluidigm, San Fransisco, CA). EQ™ four element calibration beads (Cat. No. 201078, Fluidigm, San Francisco, CA) were spiked into each sample to allow for signal normalization. Normalization was performed using CyTOF software version 6.7.1014 (Fluidigm). High dimensional data analysis was performed after identifying cell singlets (^191^Ir^+193^Ir^+^) and viable (^195^ Pt^−^) events using FlowJo Software (version 10.8.1; Becton, Dickinson & Company). All live singlet events were exported to new “.fcs” (flow cytometry standard) files prior to analysis. t-SNE clustering analysis was performed on 10,000 equivalent events from each sample with selection of all parameters. Cellular phenotypes were assigned to the t-SNE plot based on distribution and expression characteristics of all markers after clustering. Phenographs were generated in the R language-based Cytofkit software^29,30^.

### Spatial transcriptomics

Spatial transcriptomics was performed by the Center for Cell Signaling in Gastroenterology, Epigenomics and Spatial Analytics Core, Mayo Clinic, Rochester, MN, USA, on FFPE mouse liver tissue sections using the NanoString GeoMx DSP platform. Briefly, tissue sections were deparaffinized and rehydrated. Antigen retrieval was performed at 100℃ with 1X Tris EDTA (pH 9) for 15 min, followed by proteinase K (0.1 µg/ml) digestion at 37℃ for 15 min. Tissue sections were fixed for 20 min in 10% neutral buffered formalin. In situ hybridization (ISH) of mouse Whole Transcriptome Atlas (Cat No 121401103, GeoMx NGS RNA WTA Mm) was performed overnight at 37℃. Post ISH, sections were washed stringently with 1:1 saline sodium citrate (SSC)-formamide buffer. Tissue sections were labelled with morphology markers for cholangiocytes (PanCK) and macrophages^19^ (IBA-1, Clone E404W, 48934, Cell Signaling Technology). Regions of interest (ROIs) were demarcated around the ductular reaction or within the parenchyma and segmented on PanCK and IBA1 positivity (Fig S5A). Cleaved probes from segments within each ROI were transferred to separate wells of 96 well plates. The cDNA library was prepared with 18 cycles of amplification. Libraries were pooled, purified using AMPure beads and sequenced on the NextSeq2000 (Illumina) using the P2 flow cell at the Genome Analysis Core, Mayo Clinic, Rochester, MN. Quality control for data recovered from NGS sequencing was performed using set standards for alignment, trimming, and sequencing saturation. Using a custom GeoMx-NGS gene expression analysis workflow in R, segments, and genes with low signal relative to background were filtered based on limit of quantification (LOQ). Q3 normalization was performed to verify that only segments with higher than background values were included. Fold change in gene expression was calculated separately for PanCK^+^, IBA1^+^ periportal and IBA1^+ ^parenchymal macrophages based on differential expression between DDC diet and control diets within each genotype. A heatmap was created in Clustvis^31^ and volcano plots were generated in GraphPad Prism v 9.3.

### ISH for *Cxcl1*

RNA-ISH was performed on FPPE liver sections using the RNAscope 2.5 High-Definition Red Assay (Advanced Cell Diagnostics #322350) kit, following the manufacturer’s protocol. Probes targeting the mouse *Cxcl1* (#407721) were used. Following ISH, cells were blocked with the co-detection blocker (#323170), immunostained for CK19 @ 1:100 using the antibody diluent (# 323160) and detected with Alexa fluor 488 secondary antibodies. Following DAPI stain, the cells were mounted and imaged using EVOS 7000 for quantification with Image J.

### Statistical analysis

Data are expressed as mean ± S.E.M. representing replications within an experiment. Median values are plotted for violin plots. Statistical significance between multiple groups was determined by ANOVA, while statistical differences between two groups was defined by a unpaired t-test using GraphPad Prism software (v 9.3.0).

### Supplementary Information

Supplementary Information.

## Data Availability

The spatial transcriptomic datasets generated during the current study are available in the GEO database repository, GSE239667.
