# Supplementary material for: Tumor necrosis factor-related apoptosis-inducing ligand (TRAIL) deletion in myeloid cells augments cholestatic liver injury
Source: Sci Rep. 2024 Jan 25;14:2145. doi: 10.1038/s41598-024-52710-3 (PMC10810846; doi:10.1038/s41598-024-52710-3)
Supplement: Supplementary file 1 — Supplementary Information. [file 41598_2024_52710_MOESM1_ESM.docx]

Supplementary information for the article:

**Tumor Necrosis Factor-Related Apoptosis-Inducing Ligand (TRAIL) Deletion In**

**Myeloid Cells Augments Cholestatic Liver Injury**

Anuradha Krishnan^1^, Nazli Begum Ozturk^1^, Kaiyel A. Cutshaw^1^, Maria Eugenia Guicciardi^1^, Takashi Kitagataya^1^, Kirsta E. Olson^1^, Kevin D. Pavelko^2^, William Sherman^3^, Alexander Q. Wixom^3^, Nidhi Jalan-Sakrikar^1^, Michelle Baez-Faria^1^, Florencia Gutierrez^1^, and Gregory J. Gores^1^*

^1^Division of Gastroenterology and Hepatology, Mayo Clinic, Rochester, MN; ^2^Department of Immunology, Mayo Clinic, Rochester, MN; ^3^Department of Quantitative Health Sciences, Mayo Clinic, Rochester, MN.

**Table of contents:**

- Material and Methods
- Figure S1
- Figure S2
- Figure S3
- Figure S4
- Figure S5

**Materials and Methods**

**Flow cytometry**

Flow cytometry was performed on intrahepatic leucocytes and peripheral blood leucocytes isolated from WT mice fed a DDC diet for a period of 7 days. Leucocytes were stained with fluorochrome-conjugated surface markers against CD45 (Clone REA737, 130-110-796, Miltenyi Biotec), F4/80 (Clone BM8, Biolegend), Ly6G (Clone 1A8, Biolegend), and TRAIL/CD253 (Clone REA1080, 130-117-560, Miltenyi Biotec). Flow cytometry was performed on a MACSQUANT X (Miltenyi Biotec) instrument after compensation with appropriate fluorescence-minus-one (FMO) controls. The viability dye, Zombie Red (423109, Biolegend) was used to discriminate between live and dead cells. Data were analyzed on FlowJo software version 10.8.1 (FlowJo, LLC, BD Life Sciences, Ashland, OR).


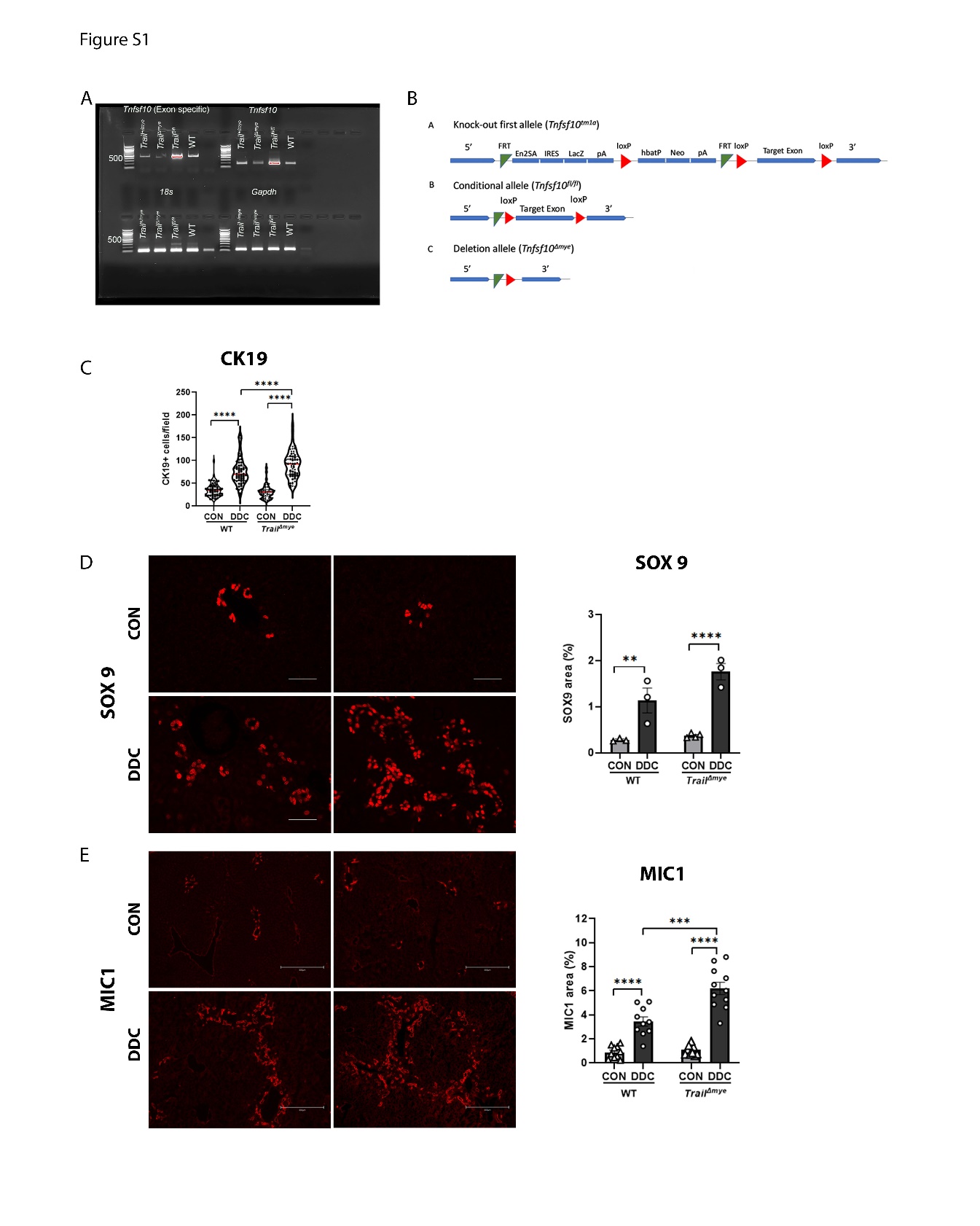


**Supplementary** **Figure S1.** **(A)** Verification of gene expression for *Trail, 18s,* and *Gapdh* in BMDM of *Trail ^+mye-^*, *Trail^∆mye +/+^, Trail^fl/fl^*, and WT mice by agarose gel electrophesis. Heterozygous mice were not included in the study. **(B)** Design of the knock-out first allele illustrating stepwise derivation of the *Trail^Δmye^* mice. **(C)** CK19^+^ cells were enumerated from at least 100 FOV for each experimental group. Data were plotted as violin plots and are indicative of the spread of ductular reaction across the liver lobule. **(D)** Representative images of FPPE mouse liver tissue sections immunostained for the ductular reaction marker, SOX 9 and **(E)** frozen mouse liver tissue sections immunostained for MIC1. Right panels provide the digital image quantification of immunostaining by area (** p < 0.01, ***-p < 0.005, **** p<0.001).


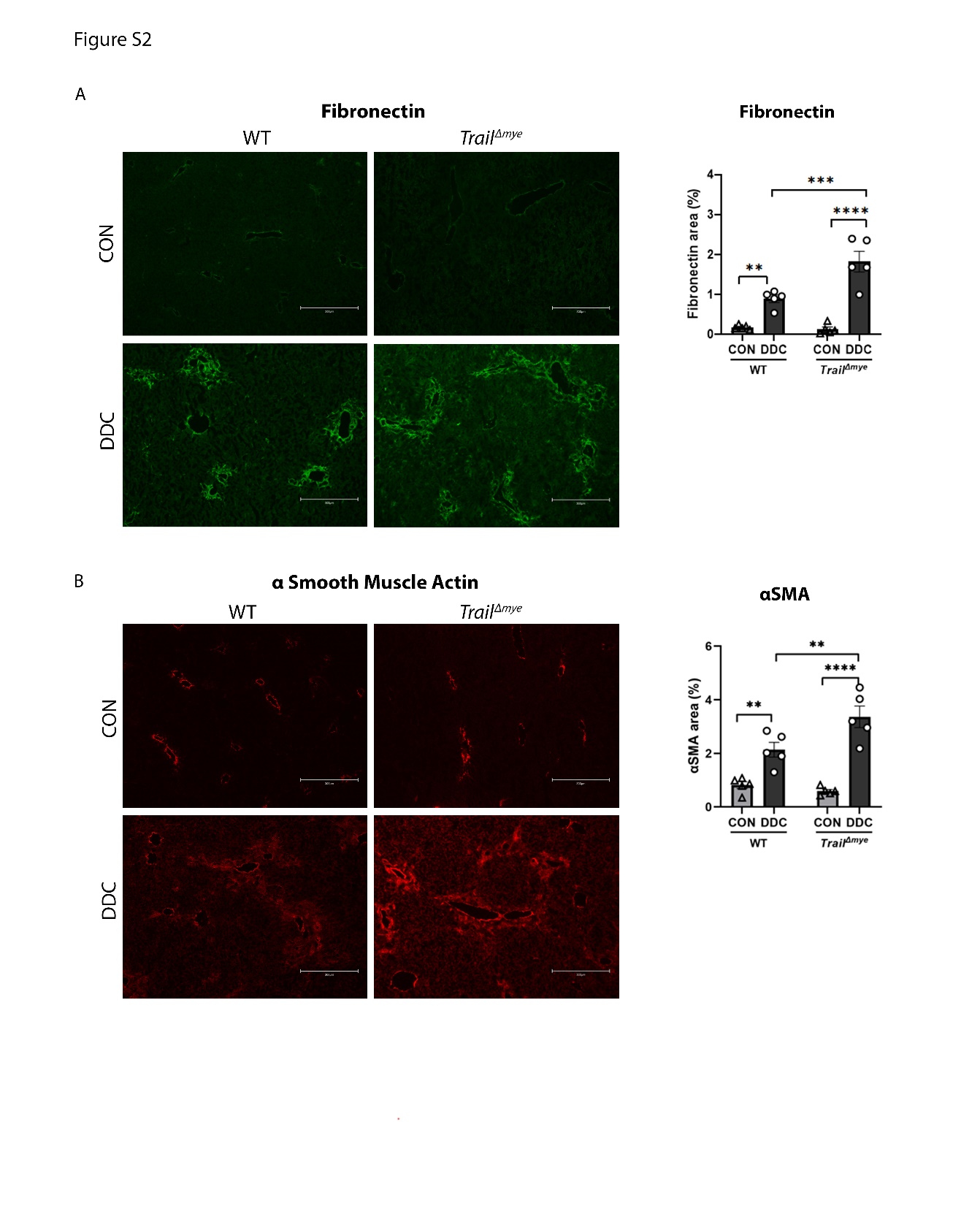


**Supplementary Figure S2. (A)** Representative images of frozen mouse liver sections immunostained for the extracellular matrix protein, fibronectin. Right panel represents the digital image analysis. **(B)** Representative images of FPPE liver tissue sections immunostained for the activated fibroblast marker, αSMA. Digital image quantification is shown in the right panel. (**p < 0.01, and ***p < 0.005, **** p<0.001).


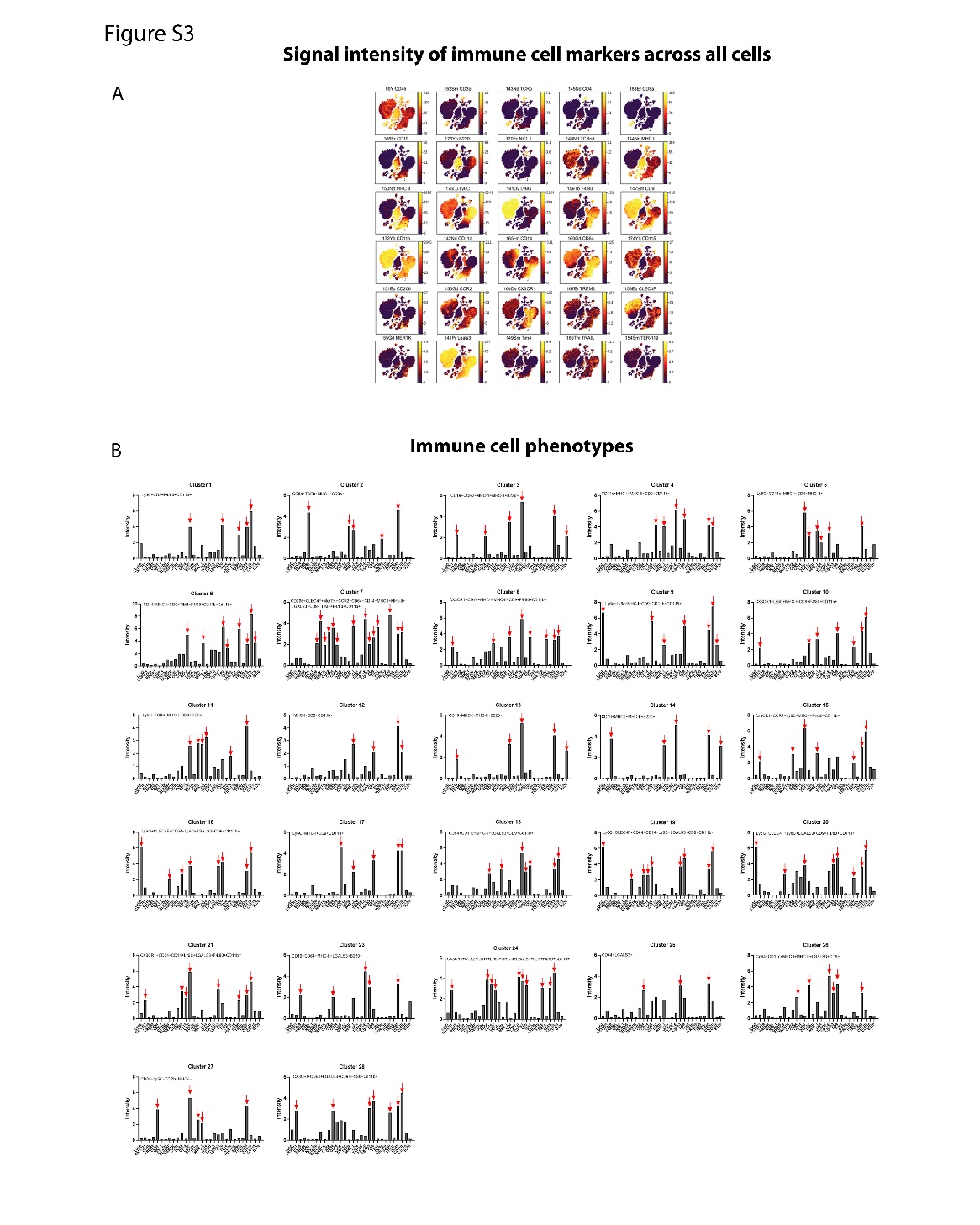


**Supplementary Figure S3. (A)** t-SNE plots illustrating signal intensity for each immune cell marker used in mass cytometry. The t-SNE plots demonstrate the distribution of signal intensity across all cells. **(B)** Mean signal intensity of immune cell marker expression is graphed for individual clusters. Red arrows identify the prominently expressed markers that define the cluster and were used for determining immune cell phenotype.


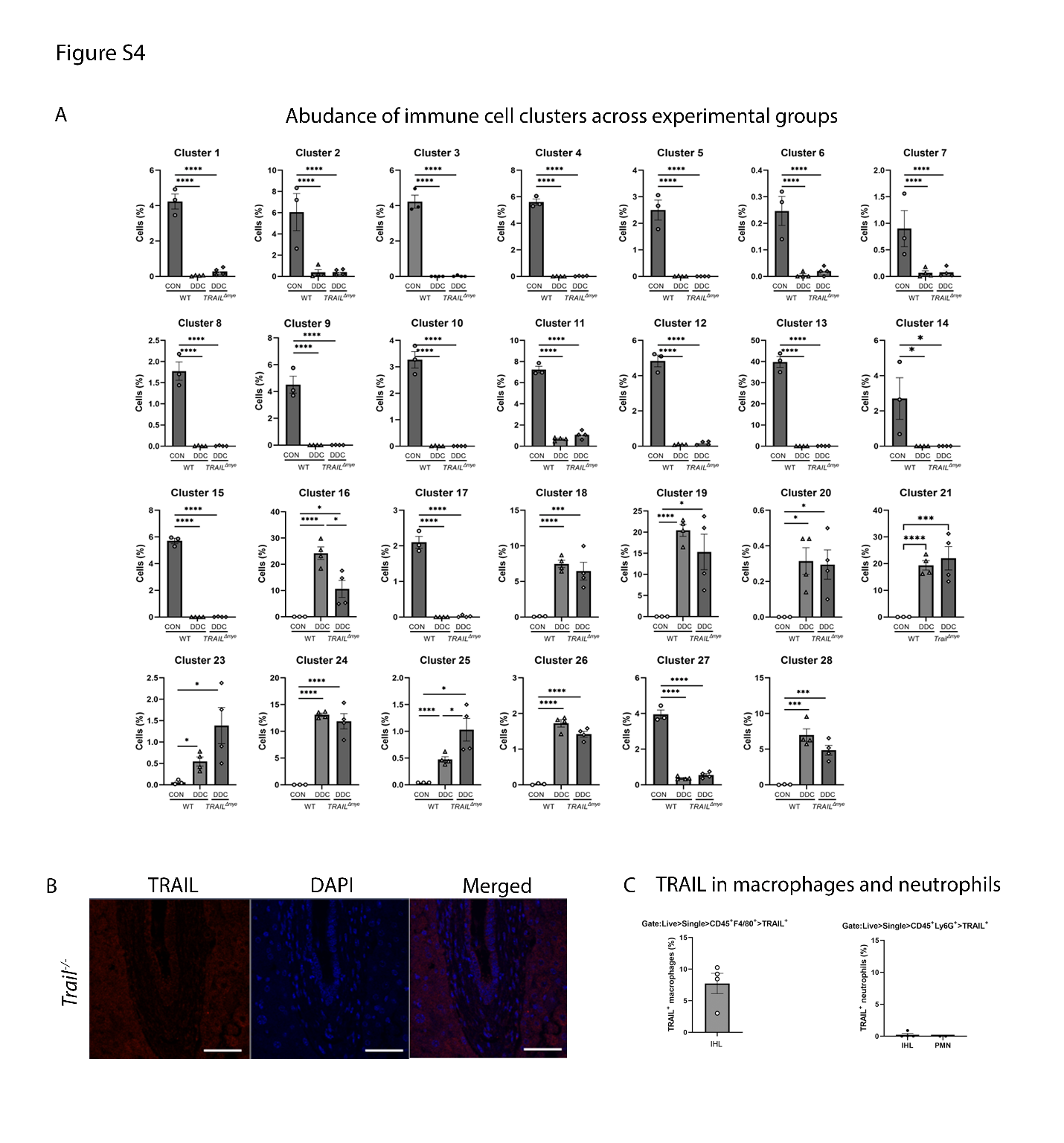


**Supplementary Figure S4. (A)** Differential abundance of individual, immune cell clusters determined by mass cytometry across the experimental groups is expressed as a percentage of intrahepatic CD45^+^ leucocytes (*p < 0.05, **p < 0.01, ***p < 0.005, **** p<0.001). **(B)**  Negative control for TRAIL immunostaining. Antibody specificity for TRAIL immunostaining was verified in the *Trail^-/-^* mouse liver tissue section. **(C)** WT mice were fed a DDC diet for a week prior to isolating intrahepatic leucocytes. TRAIL expression was determined by flow cytometry in intrahepatic macrophages (left panel), in peripheral blood neutrophils, and intrahepatic neutrophils (right panel).


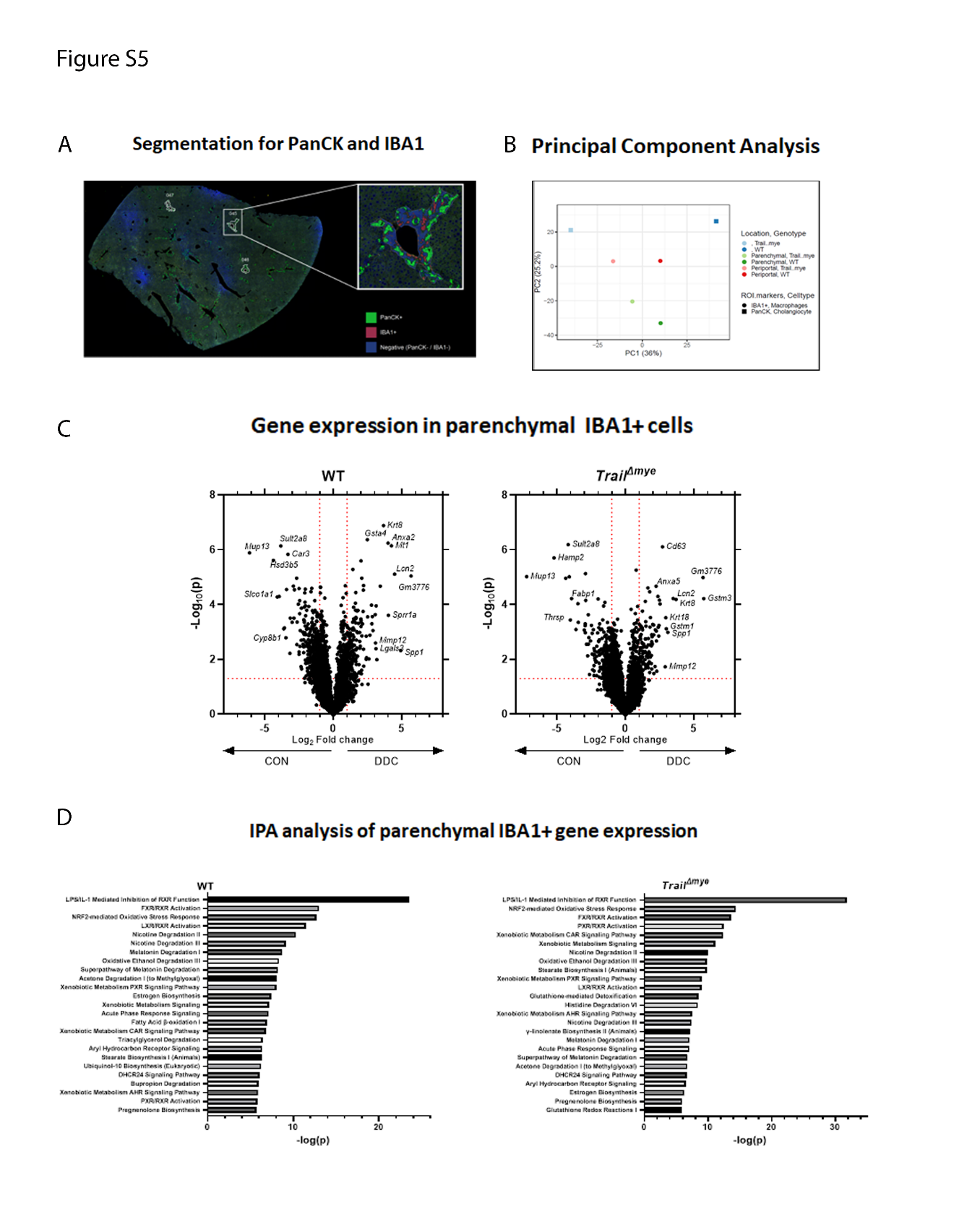


**Supplementary Figure S5. (A)** Representative image of FPPE mouse liver tissue section used for spatial transcriptomics, showing periportal regions of interest (ROI) outlined in white. Inset: A single ROI is magnified and shows the segmentation for cholangiocytes and macrophages. Following whole transcriptome hybridization, tissue sections were immunostained for PanCK^+^ (green) and IBA1^+^ (red). Segmentation allowed for the collection of PanCK^+^ and IBA1^+^ specific transcriptomes for sequencing. **(B)** Principal component analysis of PanCK^+^, IBA1^+^ periportal, and IBA1^+^ parenchymal segments are shown. **(C)** Volcano plots of differential gene expression of IBA1^+^ parenchymal segments in WT and *Trail^Δmye^* mice. **(D)** IPA analysis showing the top signaling pathways activated in the parenchymal IBA1^+^ segments of WT and *Trail^Δmye^* mice.
